# The Diagnosis and Treatment of Tuberculous Pleurisy

**Published:** 1906-03-10

**Authors:** 


### THE DIAGNOSIS AND TREATMENT OF TUBERCULOUS PLEURISY.

In dealing with this subject Dr. Sidney Martin [1] remarks on the difficulty of diagnosis between tuberculous and non-tuberculous forms of pleurisy. In many cases at the outset no positive conclusion is possible, the general symptoms and physical signs in the two groups being practically identical. The diagnosis is to be made from (1) a study of the mode of onset; (2) the course of the disease, including an examination of the fluid found in the pleura; and (3) the after-effects produced on the lung and chest wall. In both tuberculous and non-tuberculous forms of the disease the onset may be sudden, with local pain, cough, pyrexia, and dyspnœa. But in certain cases of the former the onset does not follow this type, but takes a form which is highly suggestive of tubercle. In some of these the disease begins insidiously with some pyrexia, but no physical signs or symptoms of chest disease, though sometimes there is appreciable deficiency of movement of the affected side. Then after an interval—a week or more—there are well-marked signs of pleural effusion. In a second group the tuberculous nature of the disease is suggested by a history of one or more similar attacks of pleurisy either on the same or the opposite side. Whether commencing in the "latent" form first described or in the "recurrent" fashion just mentioned, a pleurisy in all probability has tubercle as its basis.

Regarding the course and local effects of non-tuberculous pleurisy, whether with or without effusion, its tendency (excluding cases of empyema) is to get well. In the simple "dry" form it may run only a short course, and leave no local deformity of the chest wall. With effusion the pyrexia may continue 10, 14, or even 28 days, and the fluid is either absorbed, or, on removal by paracentesis, does not as a rule re-accumulate. The deformity left even by a large effusion is not great. In children no defect may be appreciable after a few weeks' convalescence, and even in adults whilst the chest movements and the breath sounds may be deficient for a time, there is no marked or permanent deformity. The fluid in non-tuberculous pleurisy as a rule coagulates spontaneously, and gives evidence of the presence of the pneumococcus, staphylococcus, or streptococcus. In tuberculous pleurisy, on the other hand, the acute stage is longer, recovery is slower, and there is a decided tendency to marked deformity of the chest. In the slighter cases there is pyrexia, showing an evening rise and morning fall, with friction sound, the latter usually in the lower part of the axilla. As the febrile process continues, defective movement, dull percussion, weak respiratory murmur, and retraction of the chest wall are noted, and the friction sound disappears. These local signs are due to thickening of the pleura, and puncture of the chest shows the absence of either serous or purulent effusion. Such cases may recover, or a subacute general tuberculosis or chronic disease of the lung may develop. When tuberculous pleurisy is associated with effusion the diagnosis from the non-tuberculous variety may be very difficult. The following considerations are of value:—(1) In tuberculous cases pyrexia may continue for weeks and months, and without the development of empyema; (2) A second, third, or even further paracentesis is usually required; (3) the fluid, when removed, sometimes coagulates spontaneously, but in long-continued cases coagulation is slight or absent. The fluid may sometimes contain the bacteria which are the cause of non-tuberculous, primary pleurisy; but if these are absent, that is, if the fluid is sterile by cultivation on ordinary media, the disease is tuberculous; or the presence of tubercle bacilli may be proved by microscopical examination or inoculation of the deposit from the centrifugalised fluid. In some few cases the tuberculous nature of a pleurisy is shown by extension of the disease to the peritoneum, as shown by the gradual appearance of tumidity of the abdomen, or by the presence of some fluid effusion. Much more common is extension to the lung or co-incident tuberculous disease of the lung as shown by the detection of tubercle bacilli in the sputa. But all cases of pleural effusion with expectoration are not tuberculous. Pneumococcal and streptococcal pleurisies may have this symptom, but here again the examination of the sputa reveals the diagnosis. As persistent effects of a tuberculous pleurisy may be seen considerable retraction of the chest wall, diminished entrance of air, defective resonance (modified, perhaps, by local compensatory emphysema), and signs of dilatation of the bronchi. These signs may persist even though the patient's general condition is that of physical well-being, and there is no expectoration.

In reference to treatment, rest in bed and good nursing are, of course, indicated. The important point is the question of paracentesis, and it may be said that there is no more danger in drawing off the fluid in tuberculous than in non-tuberculous cases. Thus, apart from considerations of urgency, as, for example, the existence of respiratory distress, it is good practice to draw off the fluid when it is large in quantity and shows no tendency to spontaneous absorption. With prolonged pyrexia and the absence of urgent symptoms the patient should rest in the open air, being at the same time well wrapped up and well fed, and open-air treatment after the pyrexia has ceased is the best method to promote healing of the tuberculous lesion and to prevent recurrences.

Lancet, March 3, 1906.

